# Ionic Liquid Mixture Electrolyte Matching Porous Carbon Electrodes for Supercapacitors

**DOI:** 10.3390/ma15207400

**Published:** 2022-10-21

**Authors:** Yuhua Zhao, Yujuan Chen, Quanzhou Du, Kelei Zhuo, Lifang Yang, Dong Sun, Guangyue Bai

**Affiliations:** Collaborative Innovation Center of Henan Province for Green Manufacturing of Fine Chemicals, Key Laboratory of Green Chemical Media and Reactions, Ministry of Education, School of Chemistry and Chemical Engineering, Henan Normal University, Xinxiang 453007, China

**Keywords:** ionic liquid mixture, electrolyte, supercapacitor, porous carbon

## Abstract

Ionic liquids (ILs), with their wide electrochemical stable potential window, are promising electrolytes for supercapacitors (SCs). The suitable matching of the ion size and shape of the ILs to the pore size and structure of porous carbon (PC) electrode materials can realize the enhanced capacitive performance of the SCs. Here we report an interesting result: The capacitance of PC-based SCs shows a quasi-sinusoidal relationship with the composition (mass fraction) of the binary IL mixture as the electrolyte. This relationship is also interpreted based on the matching between the pore sizes of the PC materials and the size/shape of various ions of the IL mixture electrolyte. This can provide a new strategy to improve the performance of SCs by formulating a suitable mixture of different ILs to match the carbon-based electrode materials with a special pore size distribution.

## 1. Introduction

Supercapacitors (SCs) are considered a type of future important energy storage device owing to their unique characteristics, such as quick charging/discharging, outstanding specific power, long cycle life, and good safety [1,2,3,4,5,6]. To improve the performance of SCs, researchers have focused on the construction of various nano-carbon electrode materials [7,8]. For example, activated carbon has been prepared widely and used for electrical double-layer capacitors (EDLCs) [9,10]. Meanwhile, room-temperature ionic liquids (ILs) have been applied as electrolytes for SCs based on their larger electrochemical stable potential window (ESPW) and non-volatility when compared with aqueous and organic electrolytes [11,12,13,14]. Therefore, much attention is currently focused on the application of IL electrolytes in carbon-based supercapacitors [15,16]. However, matching the size of IL ions to the pore size of porous carbon (PC) is a key factor to enhance the performance of SCs [17,18,19]. Gogotsi and co-workers [17,20] researched the capacitive performance of the pore size of PCs in 1.5 M 1-ethyl-3-methylimidazolium tetrafluoroborate ([Emim][BF_4_]) in acetonitrile and pure 1-butyl-3-methylimidazolium bis(trifluoromethylsulfonyl)imide ([Bmim][TFSI]), and concluded that, for a given electrolyte, the maximum specific capacitance appeared when the pore size was very close to the ion size (the ratio of the ion size to pore size is 1). Chen and co-workers took ILs’ ions as spheres in theoretical modeling and concluded that, for a given pore size distribution (PSD) of porous carbon materials, the decrease in the ion diameter results in enhanced capacitance [19]. 

In addition, the mixtures of several ILs as the electrolyte for SCs can also enhance the performance of the SCs [21]. Wang et al. [22]. found selective charging behavior of the IL mixtures with the same anion (BF_4_^−^) and different cations. Yambou et al. [23] noted that the ternary mixture of ILs with the same cation (Emim^+^) and different anions can work effectively at low temperatures in carbon-based EDLCs. Gogotsi and coworkers discovered that binary IL mixtures used as the electrolyte (binary IL electrolyte) could expand the operating potential window and increase the capacitive performance of the supercapacitors based on two identical onion-like carbon (OLC) electrodes (OLC has an open surface structure, which is free from micropores), because more counterions are accumulated on the electrode surface due to the mixing effect [24,25,26]. Obviously, it should be a potential strategy to design a mixture of ILs with different/same cations/anions as the electrolyte for high-performance SCs. Therefore, it is necessary to explore the effect of the ion composition and size of mixed IL electrolytes on the performance of porous carbon-based SCs.

This work aimed at matching the ion size of IL electrolytes to the pore sizes of a porous carbon electrode by adjusting the composition (mass fraction, *w*) of two ILs in their binary mixture to improve the performance of SCs. An interesting phenomenon has been observed: A quasi-sinusoidal curve between the specific capacitance and the mass fraction of the ILs. The relationship depends on the matching of the ion sizes to the pore sizes of PCs, as well as the consistency of their structures and shapes. Accordingly, a new strategy can be proposed to formulate the IL mixture electrolyte for high-performance energy storage devices.

## 2. Experimental Section

### 2.1. Chemicals and Materials

1-Ethyl-3-methylimidazolium tetrafluoroborate ([Emim][BF_4_], ≥99%) and1-butyl-3-methylimidazolium bis(trifluoromethylsulfonyl)imide ([Bmim][TFSI], ≥99%) were purchased from Lanzhou Institute of Chemical Physics, Chinese Academy of Sciences (Lanzhou, China). Commercial activated carbon (AC) was purchased from Beike 2D Materials Co., Ltd. (Suzhou, China). Polytetrafluoroethylene (PTFE, 60%) was provided by Aladdin Industrial Corporation (Shanghai, China). All the chemicals were used as received without further purification.

### 2.2. Binary IL Mixture Electrolytes

These two pure parent ILs were used to prepare their binary mixture electrolytes under a moisture-free atmosphere in a glove box (H_2_O, O_2_ < 0.1 ppm). All IL-electrolytes underwent oven-dried pre-treatment before use. These binary IL mixtures were marked with [Bmim][TFSI]*_w_*[Emim][BF_4_]_1−*w*_, where *w* and 1−*w* represent the mass fraction of [Bmim][TFSI] and [Emim][BF_4_] (*w* = 0, 0.2, 0.4, 0.5, 0.6, 0.8, and 1), respectively.

### 2.3. Characterization

The nitrogen adsorption and desorption isotherms of the AC material were measured on a micromeritics analyzer (ASAP 2020, Micromeritics, Norcross, GA, USA). The pore size distribution was calculated based on the Nonlocal density functional theory.

### 2.4. Preparation of Electrodes and Electrochemical Measurements

Activated carbon, acetylene black, and polytetrafluoroethylene were mixed together with a mass ratio of 85:10:5 in ethanol and then sonicated to obtain a homogeneous slurry. After that, the slurry was pasted on the nickel foam as electrodes for SCs. The detailed method was consistent with prior work [1]. The mass loading on each electrode was approximately 3 mg cm^−2^.

Cellulosic NKK TF 4030 as the separator was sandwiched between two electrodes, and [Emim][BF_4_], [Bmim][TFSI], or [Bmim][TFSI]*_w_*[Emim][BF_4_]_1−*w*_ (40 μL) was used as the electrolyte to assemble CR2032 coin-type symmetric supercapacitors in a glove box under an argon atmosphere (water content < 0.1 ppm, oxygen content < 0.1 ppm). Electrochemical performances were evaluated through cyclic voltammetry (CV), galvanostatic charge/discharge (GCD), and electrochemical impedance spectroscopy (EIS), which were measured on an electrochemical workstation (CHI660D, Shanghai Chenhua Instrument Co., Ltd., Shanghai, China) at 10−50 °C. CV tests were performed at different scan rates in the range of 10 mV s^−^^1^ to 200 mV s^−^^1^m and the GCD profiles were obtained at current densities ranging from 1 A g^−1^ to 20 A g^−1^ in a voltage range of 0−4 V (see Supporting Information). The EIS measurements were performed in a frequency range of 0.01 to 10,000 Hz with an AC amplitude of 5 mV at an open-circuit voltage.

## 3. Results and Discussion

**Figure 1:**
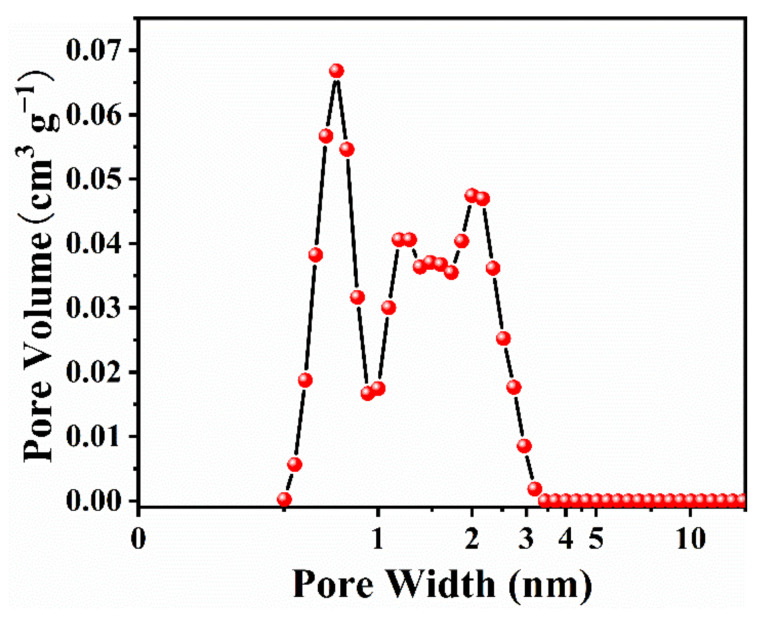
Pore size distribution of the activated carbon.

Two ionic liquids [Emim][BF_4_] and [Bmim][TFSI] were selected as parent ILs to prepare their binary mixtures ([Bmim][TFSI]*_w_*[Emim][BF_4_]_1*−w*_) at different mass fractions ranging from 0 to 1. The structure and size of ions of the two ILs are shown in Figure S1. Commercial activated carbon was employed as the electrode material in these binary IL mixture electrolytes to assemble symmetrical two-electrode devices. The structure, morphology, and pore size distribution of the activated carbon were characterized, and the results are given in Figures S1 and S2 and Figure 1. The distribution and percentage of various pore sizes and the specific surface area of the activated carbon were also calculated and are listed in Table S1. The selection of the carbon electrode material was based on the consideration of matching its porous size and distribution to the size of ions of the ionic liquid electrolyte. Through the matching relationship, we can explore how the IL mixture electrolyte enhances the capacitive performance of carbon-based supercapacitor devices by increasing the utilization rate of various pores.

**Figure 2:**
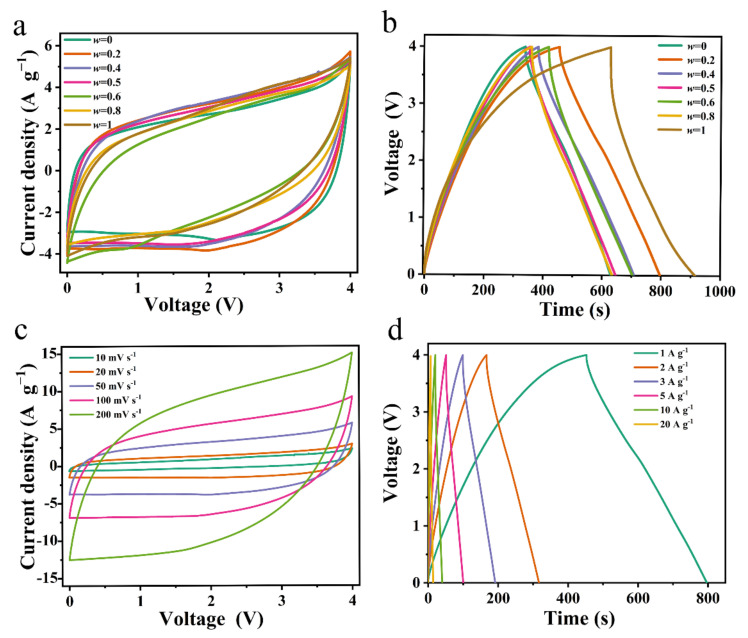
Electrochemical performance of [Bmim][TFSI]*_w_*[Emim][BF_4_]_1*-w*_ electrolyte at 30 °C: (**a**) CV curves at 50 mV S^−1^ and (**b**) GCD curves at 1 A g^−1^. (**c**) CV and (**d**) GCD curves in [Bmim][TFSI]_0.2_[Emim][BF_4_]_0.8_ electrolyte at 30 °C.

Some electrochemical performances of these constructed SCs were determined at different temperatures (10, 20, 30, 40, and 50 °C), and are shown in Figure 2, Figures S3 and S4. The rectangular shape without obvious distortions for the cyclic voltammogram (CV) curves at a scan rate of 50 mV s^−1^ (Figure 2a) and the linear galvanostatic charge/discharge (GCD) profiles (Figure 2b) indicate good capacitive behavior. The CV curves of [Bmim][TFSI]_0.8_[Emim][BF_4_]_0.2_ exhibited quasi-rectangular shapes at different scan rates (Figure 2c). The electrostatic charge storage mechanism and the excellent rate capability were suggested for [Bmim][TFSI]_0.8_[Emim][BF_4_]_0.2_ electrolytes since CV shapes still maintained quasi-rectangular shapes, even at the high scan rates. The symmetric GCD curves showed no clear evidence of the voltage drop at different current densities (Figure 2d).

**Figure 3:**
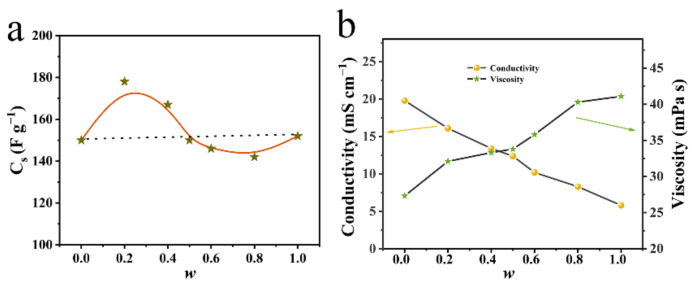
Plots of (**a**) specific capacitance and (**b**) conductivity/viscosity vs. the mass fraction *w* of the binary IL mixtures ([Bmim][TFSI]*_w_*[Emim][BF_4_]_1-*w*_) at 30 °C.

It is very interesting that we observed quasi-sinusoidal relationship curves between the specific capacitance and the mass fraction *w* of [Bmim][TFSI] in binary [Bmim][TFSI]*_w_*[Emim][BF_4_]_1−*w*_ mixtures, as shown in Figure 3a at 30 °C (as an example) and Figure S6 at all test temperatures. Figure 3a represents that the specific capacitance has a maximum value at *w* = 0.2 (i.e., [Bmim][TFSI]_0.2_[Emim][BF_4_]_0.8_), which is more than those at *w* = 0 or 1 (i.e., two pure parent ILs). Meanwhile, there is a minimum value at *w* = 0.8 (i.e., [Bmim][TFSI]_0.8_[Emim][BF_4_]_0.2_), which is less than that for both pure parent ILs.

As is well-known, the effect of the electrolyte on the SC performance includes many factors, such as viscosity, conductivity, and ionic size/shape matching with the pores of materials. To interpret the quasi-sinusoidal relationship, we measured densities, viscosities, and electric conductivities of the two pure ILs and their binary mixtures, and results are listed in Tables S2–S4. Figure S7b,c indicate the relationship of viscosity/conductivity with the mass fraction (*w*) of the IL-mixtures. Obviously, the near-linear changes of viscosity and conductivity are observed with the mass fraction (*w*) of the mixed ILs, and the values for all binary IL mixtures are located between those of these two pure ILs. Consequently, the change in viscosity and conductivity should not be the main factors that determine the quasi-sinusoidal relationship between the capacitance and the mass fraction, although they can affect the capacitive performance of the SCs.

To understand this interesting relationship, we first consider the matching of the ion sizes and shapes of IL electrolytes to the pore sizes of the electrode material. As mentioned above, the Gogotsi group [17,20,22] and the Chen group [27] theoretically and experimentally explored the influence of matching the ion size to the pore size of porous carbon electrode materials on the capacitance of SCs, and showed that for a given porous material, when the ion size approaches the value of the pore size, the specific capacitance increases anomalously.

In these two pure ILs ([Emim][BF_4_] and [Bmim][TFSI]), the specific capacitance values of the electrode material are nearly equal, and similar results were also reported in the literature [15,28]. Based on the conclusion of Chen et al. [19], the capacitance value in [Bmim][TFSI] should be much smaller than that in [Emim][BF_4_], since the ion sizes of the former are larger than the latter. However, there is a contrary result in this work. Therefore, besides the matching of ion size to pore size, the structures and shapes of ions should also be considered to explain the phenomena.

Although BF_4_^−^ (0.45 nm) has a smaller diameter than TFSI^−^ (0.79 nm), they possess different structures and shapes [29]. BF_4_^−^ is a tetrahedral structure and its negative charge center is located at the B atom, while the spatial structure of TFSI^−^ is organized in a chain overall and the negative charge center of TFSI^−^ is located at the N atom. The negatively charged N atom accesses the surface of carbon materials more easily than the B atom, and thus, when compared with BF_4_^−^, the double-layer distance (*d*) between TFSI^−^ and the carbon surface is shorter and contributed a larger EDL capacitance (*C*) according to *C* = *εA*/*d*, where *ε* is the local relative dielectric constant and *A* is the surface area.

Figure 3 shows that when a small quantity of [Bmim][TFSI] was mixed with [Emim][BF_4_] (*w* < 0.2), the *C*_s_ value increased up to the maximum (178 F g^−1^) at *w* = 0.2. This trend should be mainly ascribed to the TFSI^−^ anion: (1) Compared with TFSI^−^, smaller BF_4_^−^ would selectively enter the micropores of the carbon material (*D*/*d*_ion_ < 2, where *D* and *d*_ion_ are the pore diameter of the porous carbon and the ion diameter, respectively), since BF_4_^−^ has a weaker interaction with [Emim]^+^/[Bmim]^+^ cations when compared with TFSI^−^ [22,23,30]. (2) As mentioned above, the TFSI^−^ ion has a variable chain structure, and thus the N atom of TFSI^−^ can access the surface of the electrode in between the absorbed BF_4_^−^ anions in relatively large micropores and mesopores (*D*/*d*_ion_ > 2), which will increase the total quantity of the absorbed anions on the surface area of the positive electrode [19].

With the increase in the added quantity of [Bmim][TFSI] (*w* = 0.2 to 0.8), the *C*_s_ decreased progressively to a minimum (141 F g^−1^), which was less than those for both of the two pure ILs. This trend can be interpreted below. When more [Bmim][TFSI] was added, more TFSI^−^ anions with a larger size would also disturb the dense arrangement of BF_4_^−^ and TFSI^−^ ions in relatively large pores (> *d*_TFSI_) on the positive electrode surface to decrease the density of the adsorbed anions (negatively charged layer), resulting in a reduction of capacitance. On the other hand, partial Bmim^+^ anions would enter micropores (> *d*_Bmim_) to reduce the contribution of the micropores to the capacitance of the negative electrode. Until *w* = 0.8, the *C*_s_ value reached a minimum (141 F g^−1^). When *w >* 0.8, the property of the binary IL electrolyte was mainly determined by [Bmim][TFSI], and thus the *C*_s_ value increased gradually to the value for pure [Bmim][TFSI] (*w* = 1) upon increasing the quantity of [Emim][BF_4_].

We also note that Osti et al. reported a similar sinusoidal relationship between the excess adsorption number of [Emim]^+^ ions near an OLC and the composition (vol%) of [Emim][BF_4_] in the binary IL mixture ([Emim][TFSI] + [Emim][BF_4_]), which was derived by the computation of classical density functional theory (cDFT), and was also confirmed by experimental measurements. However, the OLC material has an open surface structure, which is free from micropores. In this work, we demonstrate that the capacitance depends on the matching of the ion size of the mixed IL electrolyte to the pore structure of the porous carbon electrode materials [26].

Besides, we have also observed that at all test temperatures from 10 °C to 50 °C, the relationship between the values of *C*_s_ and the mass fractions *w* follows a quasi-sinusoidal curve form (Figure S5). For a given *w*, the *C*_s_ value increases with increasing temperatures (Figure S5). A similar temperature−capacitance relationship has also been reported in the literature [9,17]. Both experimental and theoretical research [19,31] suggested that electrolyte ions have a smaller effective size at higher temperatures, which leads to a decrease in the distance between the absorbed ions and the charged surface of the electrode. Consequently, the *C*_s_ value will increase as the temperature rises. In addition, the value of the equivalent series resistance (ESR, including the resistances of the bulk electrolyte, the electrodes, and the contact between the electrodes and the current collectors, see Figure S6 in the Supporting Information) reduces with increasing temperatures, which is beneficial to the enhanced performance of the SCs. The dependence of the ESR on the temperature is mainly determined by the viscosity and conductivity of the mixed IL electrolytes. As shown in Figure S7, the viscosity decreases and the conductivity increases with increasing temperatures, which is conducive to ionic transport in recharging/discharging processes.

## 4. Conclusions

In summary, we used the binary IL mixture ([Emim][BF_4_] and [Bmim][TFSI]) at various mass fractions as the electrolyte to assemble porous carbon-based SCs. The experimental results show that the specific capacitance of the SCs depends on the mass fraction of the IL mixture electrolyte and indicates a quasi-sinusoidal relationship. The relationship is mainly determined by the matching of the ion size of the binary IL mixture with the pore size of porous carbon electrodes, together with the structure and shape of these ions. Particularly, at a mass fraction of *w* = 0.2 ([Emim][BF_4_]_0.2_[Bmim][TFSI]_0.8_), the supercapacitor has the maximum specific capacitance, which is larger than those of the two pure ILs as well as their mixture. Based on this discovery, it is a promising strategy to formulate a task-specific binary IL electrolyte by choosing two different ILs, which can match the pore size distribution and structure of the porous carbon electrode materials to achieve enhanced capacitive performance of energy storage devices.

## Supplementary Materials

The following supporting information can be downloaded at: https://www.mdpi.com/article/10.3390/ma15207400/s1, Table S1: Main distribution of pore size and surface area of activated carbon. Table S2: Densities (*ρ*) of neat ILs [Emim][BF_4_]/[Bmim][TFSI] and their binary mixtures [Bmim][TFSI]*_w_*[Emim][BF_4_]_1*-w*_ at different temperatures (*t*). Table S3: Viscosities (*η*) of neat ILs [Emim][BF_4_]/[Bmim][TFSI] and their binary mixtures [Bmim][TFSI]*_w_*[Emim][BF_4_]_1*-w*_ at different temperatures (*t*). Table S4: Conductivities (*σ*) of neat ILs [Emim][BF_4_]/[Bmim][TFSI] and their binary mixtures [Bmim][TFSI]*_w_*[Emim][BF_4_]_1*-w*_ at different temperatures (*t*). Figure S1: The structure and size of ions of the two ILs [Bmim][TFSI] and [Emim][BF_4_]. (HyperChem models of the structure of these ions show their sizes [29]. ^a^ Calculated by taking the ions as a sphere from ions volume [22,32]). Figure S2: Nitrogen adsorption/desorption isotherm and pore size distribution curve (inset) of the commercial activated carbon material. Figure S3: Cyclic voltammograms (50 mV s^−1^) in [Bmim][TFSI]*_w_*[Emim][BF_4_]_1*-w*_ electrolytes at different temperatures: (a) 10 °C, (b) 20 °C, (c) 40 °C, and (d) 50 °C. Figure S4: GCD at 1 A g^−1^ in [Bmim][TFSI]*_w_*[Emim][BF_4_]_1*-w*_ electrolytes at different temperatures:(a) 10 °C, (b) 20 °C, (c) 40 °C, and (d) 50 °C. Figure S5: Plots of the specific capacitance vs the mass fraction *w* of the binary IL mixtures ([Bmim][TFSI]*_w_*[Emim][BF4]_1-*w*_) at different temperatures from 10 °C to 50 °C. Figure S6: Electrochemical performance in [Bmim][TFSI]_0.2_[Emim][BF_4_]_0.8_ at different temperatures from 10 °C to 50°C. (a) Cyclic voltammograms curves at 50 mV s^−1^. (b) Galvanostatic charge/discharge curves at 1 A g^−1^. (c) EIS spectra. Figure S7: Plots of (a)density, (b) viscosity, and (c) conductivity vs. the mass fraction of the binary IL mixtures: [Bmim][TFSI]*_w_*[Emim][BF_4_]_1*-w*_ at different temperatures from 10 °C to 50 °C. References in Supplementary Materials can be found in [22,29,32,33,34,35,36,37,38,39,40].
