# Supplementary material for: Ionic Liquid Mixture Electrolyte Matching Porous Carbon Electrodes for Supercapacitors
Source: Materials (Basel). 2022 Oct 21;15(20):7400. doi: 10.3390/ma15207400 (PMC9611629; doi:10.3390/ma15207400)
Supplement: Supplementary file 1 [file materials-15-07400-s001.zip › materials-1952738-supplementary.pdf]

Supporting Information

Ionic Liquid Mixture Electrolyte Matching Porous Carbon Electrodes for Supercapacitors

Yuhua Zhao,[+] Yujuan Chen,[+] Quanzhou Du, Kelei Zhuo,* Lifang Yang, Dong Sun, and Guangyue Bai

Collaborative Innovation Center of Henan Province for Green Manufacturing of Fine Chemicals, Key Laboratory of Green Chemical Media and Reactions, Ministry of Education, School of Chemistry and Chemical Engineering, Henan Normal University, Xinxiang, Henan 453007, China.

E-mail: kzhuo@htu.edu.cn

[+] These authors contributed equally to this work.

1. Physico-chemical characterization of binary IL mixture electrolytes

The density (ρ) of the neat ILs and their binary mixtures was measured at different temperatures from 10 °C to 50 °C (± 0.02 °C) using a density meter (Anton Paar, DMA 4500 M). The viscosity and electrical conductivity of the samples at different temperatures (10 °C − 50 °C) were also measured using a viscosimeter (Brookfield DV-II^+ Pro) and conductivity meter (Seven Compact TM), respectively.

2. Electrochemical stability windows of the binary IL mixture electrolytes

Electrochemical stability windows of the binary IL mixture electrolyte were determined on the basis of the method suggested by Weingarth et al [1].

3. Calculation method

According to the following equations, the gravimetric specific capacitance (C_s, F g^{-1}) of a single electrode, and specific capacitance (C_{cell}, F g^{-1}), energy density (E, Wh kg^{-1}), and power density (P, W kg^{-1}) of the symmetric supercapacitors were calculated from the GCD curves.

$$C_s = \frac{2I\Delta t}{m\Delta U} \tag{S1}$$

$$C_{cell} = \frac{1}{4} C_s \tag{S2}$$

$$E = \frac{1}{28.8} C_s \Delta U^2 \tag{S3}$$

$$P = 3600 \frac{E}{\Delta t} \tag{S4}$$

where I (A) is the discharge current, Δt (s) is the discharge time, ΔU (V) is the operating voltage (excluding the voltage drop), and m (g) is the mass loading of the active material on one electrode.

4. Supplementary Tables and Figures

Table S1. Main distribution of pore size and surface area of activated carbon

Pore size range/nm	Pore volume/cm^3 g^{-1}	Percentage of cumulative pore volume/%	Surface area /m^2 g^{-1}	Percentage of cumulative surface area/%
0.50~ 0.73	0.19	24	554	39
0.73~2.00	0.46	58	750	53
2.00~3.18	0.14	18	114	8

Table S2. Densities (ρ) of neat ILs [Emim][BF_4]/[Bmim][TFSI] and their binary mixtures [Bmim][TFSI]$_w$[Emim][BF_4]$_{1-w}$ at different temperatures (t)

t/°C	ρ/g·cm^{-3} at different mass fraction w								
	0	0.2	0.4	0.5	0.6	0.8	1	0	1
10	1.291	1.320	1.351	1.370	1.391	1.417	1.450		
20	1.284	1.312	1.342	1.362	1.382	1.408	1.441	1.284[a]	1.4409[c]
30	1.276	1.304	1.334	1.353	1.373	1.398	1.431	1.272[b]	1.4316[c]
40	1.268	1.296	1.326	1.345	1.365	1.389	1.421	1.268[b]	1.4221[c]
50	1.261	1.288	1.318	1.336	1.356	1.380	1.411	1.258[b]	1.4126[c]

The estimated relative standard uncertainty of experimental densities was less than 0.1%. (Please refer to the previous literature of our group.[2]). [a] Taken from ref. [3]. [b] Taken from ref. [4]. [c] Taken from ref. [5].

Table S3. Viscosities (η) of neat ILs [Emim][BF_4]/[Bmim][TFSI] and their binary mixtures [Bmim][TFSI]$_w$[Emim][BF_4]$_{1-w}$ at different temperatures (t)

t/°C	η/mPa s at different mass fraction w								
	0	0.2	0.4	0.5	0.6	0.8	1	0	1
10	60.97	80.88	83.53	88.91	94.11	99.76	105.20		
20	40.95	49.95	51.44	53.09	56.63	60.24	61.73	41.2[a]	62.08[c]
30	27.32	32.10	33.31	33.80	35.81	40.29	41.10	27.083[b]	41.24[c]
40	16.34	21.64	22.85	23.57	24.34	27.51	28.12		28.28[c]
50	12.17	15.25	16.41	17.06	18.25	19.57	20.13		20.64[c]

The estimated relative standard uncertainty of experimental viscosity was less than 0.2%. (Please refer to the previous literature of our group.[2]). [a] Taken from ref. [3]. [b] Taken from ref. [6]. [c] Taken from ref. [7].

Table S4. Conductivities (σ) of neat ILs [Emim][BF_4]/[Bmim][TFSI] and their binary mixtures [Bmim][TFSI]$_w$[Emim][BF_4]$_{1-w}$ at different temperatures (t)

t/°C	σ/ mS cm^{-1} at different mass fraction w								
	0	0.2	0.4	0.5	0.6	0.8	1	0	1
10	9.9	8.3	6.5	6.4	5.8	3.6	2.5		
20	14.8	11.6	9.8	9.6	9.0	4.8	3.6	12.16[a]	3.37[b]
30	19.8	16.1	13.4	13.2	10.2	8.3	5.8		4.72[b]
40	26.3	23.0	18.2	18.1	13.4	10.2	7.5		6.839[c]
50	34.0	26.7	24.2	23.1	17.7	12.3	9.9		9.24[d]

The estimated relative standard uncertainty of experimental conductivities was less than 0.5%.
[a] Taken from ref. [3]. [b] Taken from ref. [7]. [c] Taken from ref. [8]. [d] Taken from ref. [9].

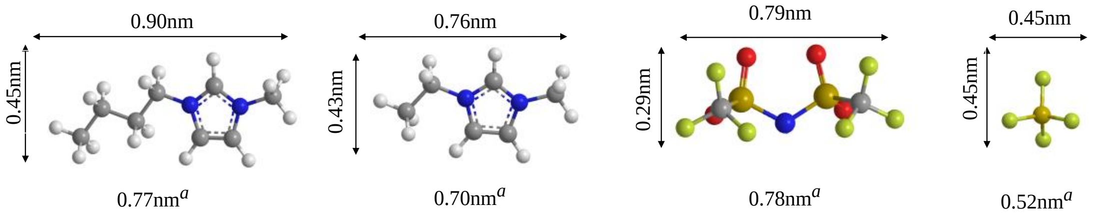

Figure S1. The structure and size of ions of the two ILs [Bmim][TFSI] and [Emim][BF_4]. HyperChem models of the structure of these ions show their sizes [10]. [a] Calculated by taking the ions as a sphere from ions volume [11,12].

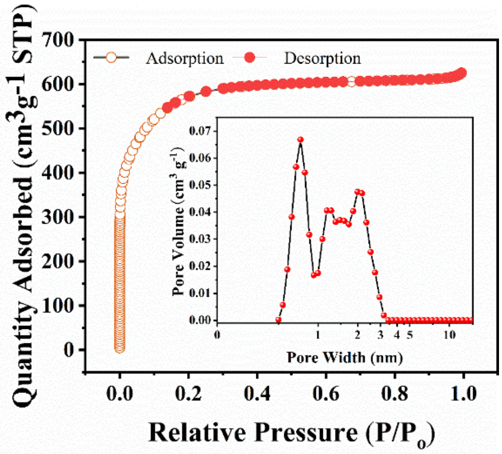

Figure S2. Nitrogen adsorption/desorption isotherm and pore size distribution curve (inset) of the commercial activated carbon material.

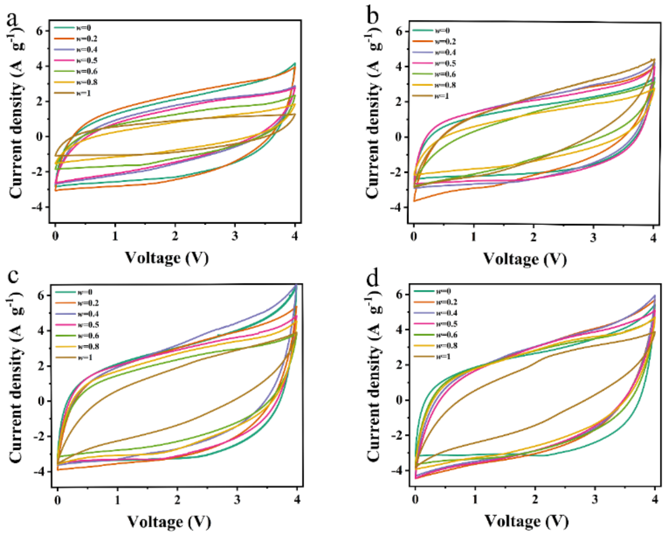

Figure S3. Cyclic voltammograms (50 mV s^{-1}) in $[Bmim][TFSI]_w[Emim][BF_4]_{1-w}$ electrolytes at different temperatures: (**a**) 10 °C, (**b**) 20 °C, (**c**) 40 °C, and (**d**) 50 °C.

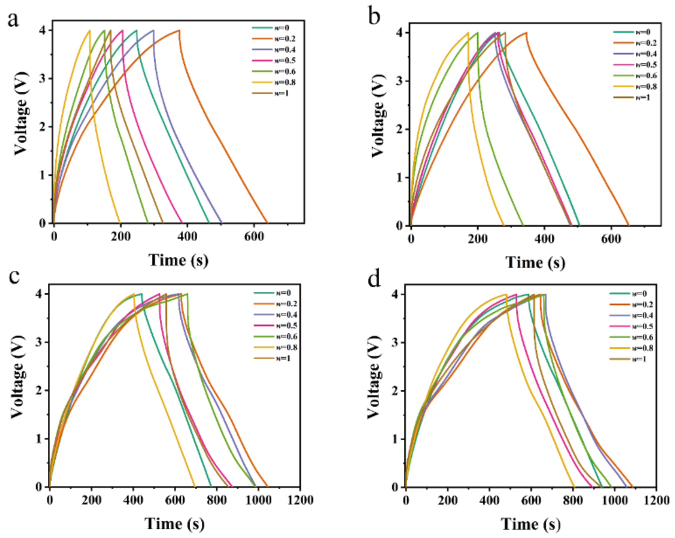

Figure S4. GCD at 1 A g^{-1} in $[Bmim][TFSI]_w[Emim][BF_4]_{1-w}$ electrolytes at different temperatures:(**a**) 10 °C, (**b**) 20 °C, (**c**) 40 °C, and (**d**) 50 °C.

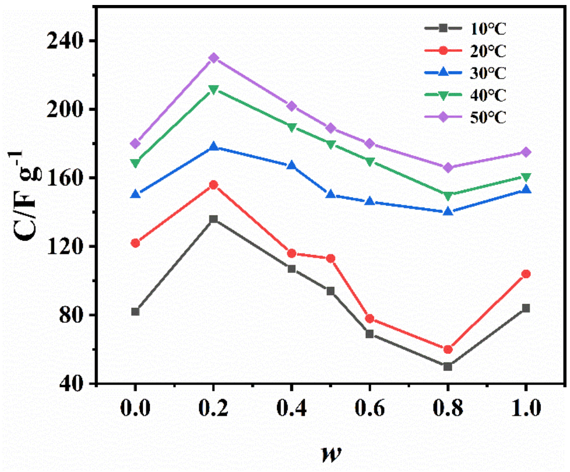

Figure S5. Plots of the specific capacitance vs the mass fraction *w* of the binary IL mixtures ([Bmim][TFSI]$_w$[Emim][BF_4]$_{1-w}$) at different temperatures from 10 °C to 50 °C.

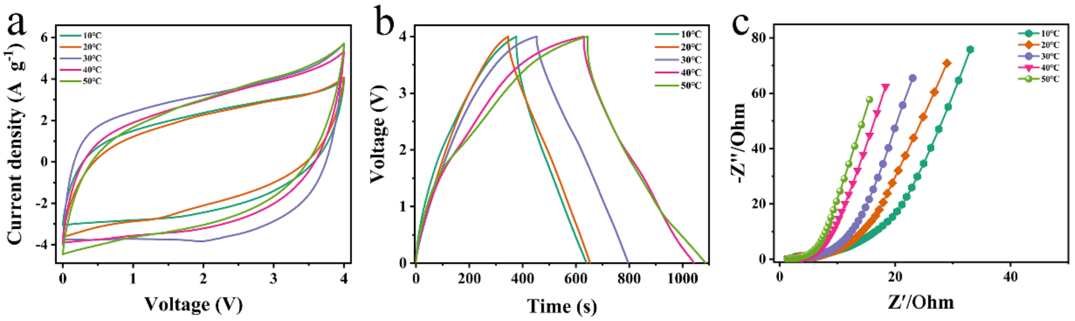

Figure S6. Electrochemical performance in [Bmim][TFSI]$_{0.2}$[Emim][BF4]$_{0.8}$ at different temperatures from 10 °C to 50°C. (**a**) Cyclic voltammograms curves at 50 mV s^{-1}. (**b**) Galvanostatic charge/discharge curves at 1 A g^{-1}. (**c**) EIS spectra.

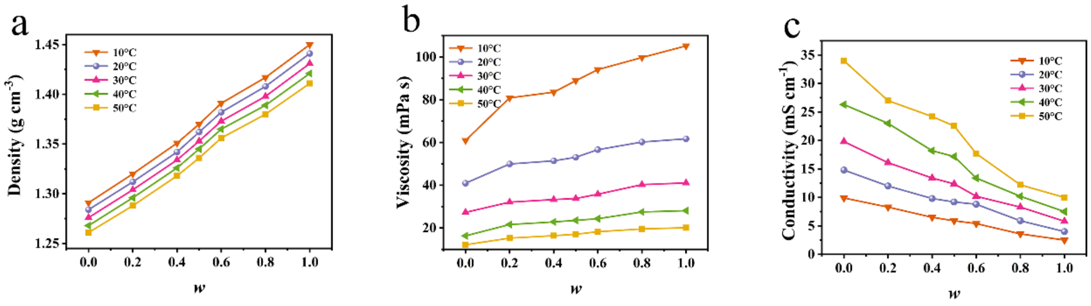

Figure S7. Plots of (**a**) density, (**b**) viscosity, and (**c**) conductivity vs. the mass fraction of the binary IL mixtures: [Bmim][TFSI]$_w$[Emim][BF_4]$_{1-w}$ at different temperatures from 10 °C to 50 °C.

References

1. Weingarth, D.; Noh, H.; Foelske-Schmitz, A.; Wokaun, A.; Kötz, R., A reliable determination method of stability limits for electrochemical double layer capacitors. *Electrochim. Acta* **2013,** *103*, 119-124. https://doi.org/10.1016/j.electacta.2013.04.057.
2. Chen, Y.; Zhuo, K.; Chen, J.; Bai, G., Volumetric and viscosity properties of dicationic ionic liquids in (glucose + water) solutions at T = 298.15 K. *J. Chem. Thermodynamics* **2015,** *86*, 13-19. https://doi.org/10.1016/j.jct.2015.02.017.
3. Yambou, E. P.; Gorska, B.; Béguin, F., Binary mixtures of ionic liquids based on EMIm cation and fluorinated anions: physico-chemical characterization in view of their application as low-temperature electrolytes. *J. Mol. Liq.* **2020,** *298*. https://doi.org/10.1016/j.molliq.2019.111959.
4. Fan, X.-H.; Chen, Y.-P.; Su, C.-S., Densities and viscosities of binary liquid mixtures of 1-ethyl-3-methylimidazolium tetrafluoroborate with acetone, methyl ethyl ketone, and N -methyl-2-pyrrolidone. *J. Taiwan Inst Chem E.* **2016,** *61*, 117-123. https://doi.org/10.1016/j.jtice.2016.01.004.
5. Hiraga, Y.; Kato, A.; Sato, Y.; Smith, R. L., Densities at Pressures up to 200 MPa and Atmospheric Pressure Viscosities of Ionic Liquids 1-Ethyl-3-methylimidazolium Methylphosphate, 1-Ethyl-3-methylimidazolium Diethylphosphate, 1-Butyl-3-methylimidazolium Acetate, and 1-Butyl-3-methylimidazolium Bis(trifluoromethylsulfonyl)imide. *J. Chem. Eng. Data.* **2015,** *60* (3), 876-885. https://doi.org/10.1021/je5009679.
6. Shamsipur, M.; Beigi, A. A. M.; Teymouri, M.; Pourmortazavi, S. M.; Irandoust, M., Physical and electrochemical properties of ionic liquids 1-ethyl-3-methylimidazolium tetrafluoroborate, 1-butyl-3-methylimidazolium trifluoromethanesulfonate and 1-butyl-1-methylpyrrolidinium bis(trifluoromethylsulfonyl)imide. *J. Mol. Liq.* **2010,** *157* (1), 43-50. https://doi.org/10.1016/j.molliq.2010.08.005.
7. Vraneš, M.; Papović, S.; Tot, A.; Zec, N.; Gadžurić, S., Density, excess properties, electrical conductivity and viscosity of 1-butyl-3-methylimidazolium bis(trifluoromethylsulfonyl)imide+γ-butyrolactone binary mixtures. *J. Chem. Thermodyn* **2014,** *76*, 161-171. https://doi.org/10.1016/j.jct.2014.03.025.
8. Vranes, M.; Dozic, S.; Djeric, V.; Gadzuric, S., Physicochemical Characterization of 1-Butyl-3-methylimidazolium and 1-Butyl-1-methylpyrrolidinium Bis(trifluoromethylsulfonyl)imide. *J.*

Chem. Eng. Data **2012,** *57* (4), 1072-1077. https://doi.org/10.1021/je2010837.

9. Widegren, J. A.; Saurer, E. M.; Marsh, K. N.; Magee, J. W., Electrolytic conductivity of four imidazolium-based room-temperature ionic liquids and the effect of a water impurity. *J. Chem. Thermodyn* **2005,** *37* (6), 569-575. https://doi.org/10.1016/j.jct.2005.04.009.
10. Jackel, N.; Kruner, B.; Van Aken, K. L.; Alhabeb, M.; Anasori, B.; Kaasik, F.; Gogotsi, Y.; Presser, V., Electrochemical in Situ Tracking of Volumetric Changes in Two-Dimensional Metal Carbides (MXenes) in Ionic Liquids. *ACS Appl. Mater Interfaces* **2016,** *8* (47), 32089-32093. https://doi.org/10.1021/acsami.6b11744.
11. Wang, X.; Mehandzhiyski, A. Y.; Arstad, B.; Van Aken, K. L.; Mathis, T. S.; Gallegos, A.; Tian, Z.; Ren, D.; Sheridan, E.; Grimes, B. A.; Jiang, D. E.; Wu, J.; Gogotsi, Y.; Chen, Selective Charging Behavior in an Ionic Mixture Electrolyte-Supercapacitor System for Higher Energy and Power. *J. Am. Chem. Soc.* **2017,** *139* (51), 18681-18687. https://doi.org/10.1021/jacs.7b10693.
12. Ye, C.; Shreeve, J. n. M., Rapid and Accurate Estimation of Densities of Room-Temperature Ionic Liquids and Salts. *J. Phys. Chem. A.* **2007,** *111* (8), 1456-1461. https://doi.org/10.1021/jp066202k.
